# Evaluating the Mechanisms of Improved Glucose Homeostasis after Bariatric Surgery in Ossabaw Miniature Swine

**DOI:** 10.1155/2014/526972

**Published:** 2014-08-24

**Authors:** Jonathan G. Sham, Vlad V. Simianu, Andrew S. Wright, Skye D. Stewart, Mouhamad Alloosh, Michael Sturek, David E. Cummings, David R. Flum

**Affiliations:** ^1^Department of Surgery, University of Washington, Seattle, WA 98195, USA; ^2^Department of Cellular and Integrative Physiology, Indiana University School of Medicine, Indianapolis, IN 46202, USA; ^3^Department of Medicine, University of Washington, Seattle, WA 98195, USA; ^4^Department of Health Services, University of Washington, Seattle, WA 98195, USA

## Abstract

*Background*. Roux-en-Y gastric bypass (RYGB) is the most common bariatric operation; however, the mechanism underlying the profound weight-independent effects on glucose homeostasis remains unclear. Large animal models of naturally occurring insulin resistance (IR), which have been lacking, would provide opportunities to elucidate such mechanisms. Ossabaw miniature swine naturally exhibit many features that may be useful in evaluating the anti diabetic effects of bariatric surgery. *Methods*. Glucose homeostasis was studied in 53 Ossabaw swine. Thirty-two received an obesogenic diet and were randomized to RYGB, gastrojejunostomy (GJ), gastrojejunostomy with duodenal exclusion (GJD), or Sham operations. Intravenous glucose tolerance tests and standardized meal tolerance tests were performed prior to, 1, 2, and 8 weeks after surgery and at a single time-point for regular diet control pigs. *Results*. High-calorie-fed Ossabaws weighed more and had greater IR than regular diet controls, though only 70% developed IR. All operations caused weight-loss-independent improvement in IR, though only in pigs with high baseline IR. Only RYGB induced weight loss and decreased IR in the majority of pigs, as well as increasing AUC_insulin_/AUC_glucose_. *Conclusions*. Similar to humans, Ossabaw swine exhibit both obesity-dependent and obesity-independent IR. RYGB promoted weight loss, IR improvement, and increased AUC_insulin_/AUC_glucose_, compared to the smaller changes following GJ and GJD, suggesting a combination of upper and lower gut mechanisms in improving glucose homeostasis.

## 1. Introduction

Roux-en-Y gastric bypass (RYGB) has emerged as the most efficient and effective approach for weight loss and treatment of type 2 diabetes mellitus (T2DM) in obese patients [1, 2]. Postoperative effects on glycemic control and insulin resistance have been shown to precede significant weight reduction [3] and occur at least partially in a weight-independent fashion [4], generating controversy surrounding the mechanisms of RYGB's antidiabetic effects [5]. Hypotheses include the operation's impact on intestinal hormones [6, 7], bile acids [8, 9], gut flora [10, 11], and intestinal glucose sensing [12] and metabolism [13]; however, no definitive mechanism has emerged.

To evaluate potential mechanisms of bariatric surgery, an animal model that mimics human metabolic disease and possesses enough anatomic similarity to allow the evaluation of bariatric procedures used in humans would be helpful [14]. Ossabaw miniature swine appear to be a valuable model of acquired obesity and IR [15]. We hypothesized that these animals would facilitate the study of RYGB mechanisms in ways not feasible in toxin-induced, rodent, or other large animal models of diabetes or insulin resistance [16]. Due to harsh environmental pressures in their native habitat, Ossabaws evolved over time to gain large amounts of weight when exposed to abundant supplies of food, reflecting selection pressure from frequent periods of famine [15]. When exposed to a high-fat, high-calorie diet in a laboratory, they develop marked obesity and many of the hallmarks of metabolic syndrome, including insulin resistance (IR) [15, 17–20], dyslipidemia [18, 20, 21], and hypertension [18, 20].

Our group has suggested several hypotheses pertaining to the antidiabetic mechanisms of various gastrointestinal (GI) rearrangements, particularly RYGB [22–31]. Among these is the “lower intestinal hypothesis,” which postulates that enhanced delivery of ingested nutrients to the distal bowel increases secretion of the incretin glucagon-like peptide-1 (GLP-1). GLP-1 elevations would augment insulin secretion and improve glucose homeostasis. The “upper intestinal hypothesis” suggests that exclusion of nutrient flow from the proximal small bowel exerts direct antidiabetes effects on glucose homeostasis, most likely by promoting a factor that increases insulin sensitivity or antagonizing a factor that decreases sensitivity. Roux-en-Y gastric bypass, gastrojejunostomy (GJ), and gastrojejunostomy with duodenal exculsion (GJD) are GI operations that facilitate the study of these hypotheses. Gastrojejunostomy enables nutrients from the stomach to be delivered directly to the distal small bowel, GJD does the same while also preventing nutrient delivery to the duodenum and proximal jejunum, and RYGB provides both proximal exclusion and enhanced distal delivery while also largely eliminating gastric nutrient exposure.

This study aims to evaluate Ossabaw miniature swine as a large animal model for metabolic syndrome and to assess the effects of several GI rearrangements on body weight and IR, potentially elucidating RYGB's influence on glucose homeostasis.

## 2. Materials and Methods

All experimental procedures involving animals were approved by the Institutional Animal Care and Use Committee at the University of Washington (UW), with the recommendations outlined by the National Research Council and the American Veterinary Medical Association Panel on Euthanasia [32, 33].

### 2.1. Animals and Environment

53 female Ossabaw swine were selected from the Indiana University School of Medicine (IUSM) Purdue Animal Facility and maintained on a standard diet of 2400 kcal/day (5L80; Purina Test Diet, Richmond, IN). A subset of 32 pigs, 12–18-month-old, were fed a high-calorie (4700 kcal/day), high-fat diet (KT324, Purina Test Diet, Richmond, IN) for ~180 days to promote weight gain and IR and were then shipped to UW. Pigs were housed in a temperature-controlled room on a 12-hour light/dark cycle with free access to drinking water and were removed daily for stall cleaning. While at the UW, pigs were maintained on a ~7500 kcal/day diet comprised of 16.1% proteins, 43.1% lipids, and 40.8% carbohydrates (5B4L, Purina Test Diet) as previously described in detail [20]. Food was provided twice daily, and intake was recorded daily. Body weight was monitored at least weekly using a digital scale (Waypig 15, Vittetoe Inc., Keota, IA).

### 2.2. Surgical Intervention

Approximately one week after pigs arrived at the UW facility, indwelling central venous access was obtained [15, 34] for repeated blood sampling as well as fluid/drug administration. After implantation of intravascular catheters, pigs were given 5–10 mg/kg of aspirin daily to reduce blood clotting around the internal catheter tip. Additionally, catheters were flushed daily with a heparin-saline solution containing 1 mg/mL vancomycin to prevent thrombosis and/or occlusion.

Approximately one month after arriving at UW, pigs were randomized to one of four GI operations, all performed in a standardized fashion: RYGB (*n* = 13), GJ (*n* = 10), GJD (*n* = 7), and Sham (*n* = 2). RYGB, which was developed in this animal model by our group [16], creates a small, functional gastric pouch measuring approximately 5 × 5 cm comprised of the proximal stomach and completely separated from the cardia, fundus, and body of the stomach. The Roux-en-Y small bowel reconstruction approximates a typical human RYGB in that ~1/3 of the small bowel is used for an alimentary Roux limb, and the biliary-pancreatic-duodenal (BPD) limb measures ~45 cm. GJ includes anastomosing the greater curvature of the stomach with the proximal jejunum, with full preservation of the stomach and pylorus. GJD is performed identically to GJ but with the additional detachment of the pylorus from the proximal duodenum using a surgical stapler, thereby excluding the duodenum from nutrient flow. In both GJ and GJD, the excluded length of small bowel is approximately the same length as the BPD limb in the RYGB procedure. The Sham operation includes an extended midline laparotomy with manual intestinal manipulation for ~130 minutes, the average time of the other procedures.

### 2.3. Glucose Tolerance and Insulin Sensitivity Testing

An intravenous glucose tolerance test (IVGTT) was performed on all pigs and within one week prior to surgery as a baseline. The IVGTT was then repeated in operated animals at 2 weeks and 8 weeks postoperatively. A standardized meal tolerance test (MTT) was performed on the day following each IVGTT in each pig. During IVGTT, after taking a baseline blood sample, 1 g/kg dextrose was administered intravenously, with subsequent venous blood sampling 5, 10, 20, 30, 40, 60, 90, and 120 min after injection. For MTT, pigs were given a test meal of 430 g of chow and allowed to eat for 15 minutes. Blood was sampled at –15, 0, 30, 60, 90, and 120 minutes after the completion of the test meal. Blood samples were evaluated at the UW's Northwest Lipid Metabolism and Diabetes Research Laboratory. Glucose levels were evaluated on a Hitachi Clinical Chemistry modular autoanalyzer (Hitachi Clinical, Tokyo), while insulin tests were performed using a Tosoh 1800 autoanalyzer (Tosoh Bioscience, San Francisco). Preoperative insulin resistance was evaluated from fasting blood samples using the homeostatic model assessment of insulin resistance (HOMA-IR) [35] using the formula HOMA-IR = (glucose × insulin)/405, where glucose and insulin are measured in mg/dL. Insulin resistance in this model was defined as a HOMA-IR >2 standard deviations from the mean for the 21 pigs that remained at the IUSM/Purdue Animal Facility and were not exposed to the high-fat, high-calorie diet.

### 2.4. Statistical Analysis

All numeric data are expressed as the average value ± the standard deviation, unless otherwise indicated. Area-under-the-curve (AUC) values were calculated using the trapezoidal approximation formula (*h*/2)∑_*k*=1_^*N*^(*f*(*x*_*k*+1_) + *f*(*x*_*k*_)), where *h* is serum insulin or glucose, *x* is time in minutes, and *k* and *N* are the lower and upper bounds of summation, respectively. Excel (version 12.3.6, Microsoft) was used for statistical analysis. Where appropriate, a paired, two-tailed, Student's *t*-test was used, with a *P* value of less than 0.05 considered statistically significant.

## 3. Results

### 3.1. Procedures

Twenty-one pigs were not fed the high-calorie diet and underwent IVGTT as regular diet controls. Thirty-two pigs were fed the obesogenic diet and subsequently randomized into one of the following groups: RYGB (*n* = 13), GJ (*n* = 10), GJD (*n* = 7), or Sham (*n* = 2). Of these, nine pigs were unable to complete the study due to perioperative complications, including intraoperative cardiac arrest (*n* = 3), postoperative infection (*n* = 3), anastomotic dehiscence (*n* = 2), and intraoperative hemorrhage (*n* = 1). Data were analyzed only for pigs that completed all three postoperative IVGTT and MTT (*n* = 23), resulting in the following cohort sizes: RYGB (*n* = 7), GJ (*n* = 8), GJD (*n* = 6), and Sham (*n* = 2).

### 3.2. Distribution of Insulin Resistance

**Figure 1:**
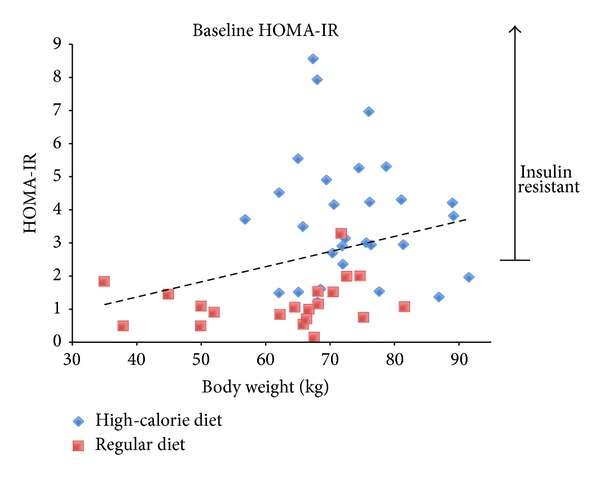
Preoperative body weight and HOMA-IR in high-calorie and regular diet Ossabaws. Scatter plot of preoperative weight and HOMA-IR in pigs exposed to the high-fat, high-calorie diet for >180 days and those maintained on a normal diet. The dashed regression line shows overall mild positive correlation between weight and HOMA-IR (*R* ^2^ = 0.08, *P* = 0.05). Insulin resistance in this model was defined as HOMA-IR > 2 standard deviations above the regular diet group mean (>2.61).

The distribution of pig body weight and HOMA-IR (Figure 1) demonstrates high variability in the relationship between increasing body weight and IR. High-calorie fed Ossabaws weighed more (73.4 versus 62.3 kg, *P* = 0.002) and had higher average HOMA-IRs, with a much wider distribution (3.7 ± 1.9) than did their regular chow-fed counterparts (1.2 ± 0.7, *P* < 0.001). The wide distribution resulted in approximately 70% of high-calorie fed pigs developing insulin resistance, as we defined it (i.e., HOMA-IR >2 standard deviations above the mean for pigs on a regular diet or 2.61. Overall, there was a mild positive correlation between body weight and HOMA-IR (Figure 1 regression line, *R* ^2^ = 0.08, *P* = 0.05). However, there was significant heterogeneity, particularly in the high-calorie group, and the heaviest animals were not necessarily the most insulin resistant.

### 3.3. Postoperative Decreases in Body Weight and HOMA-IR

**Figure 2:**
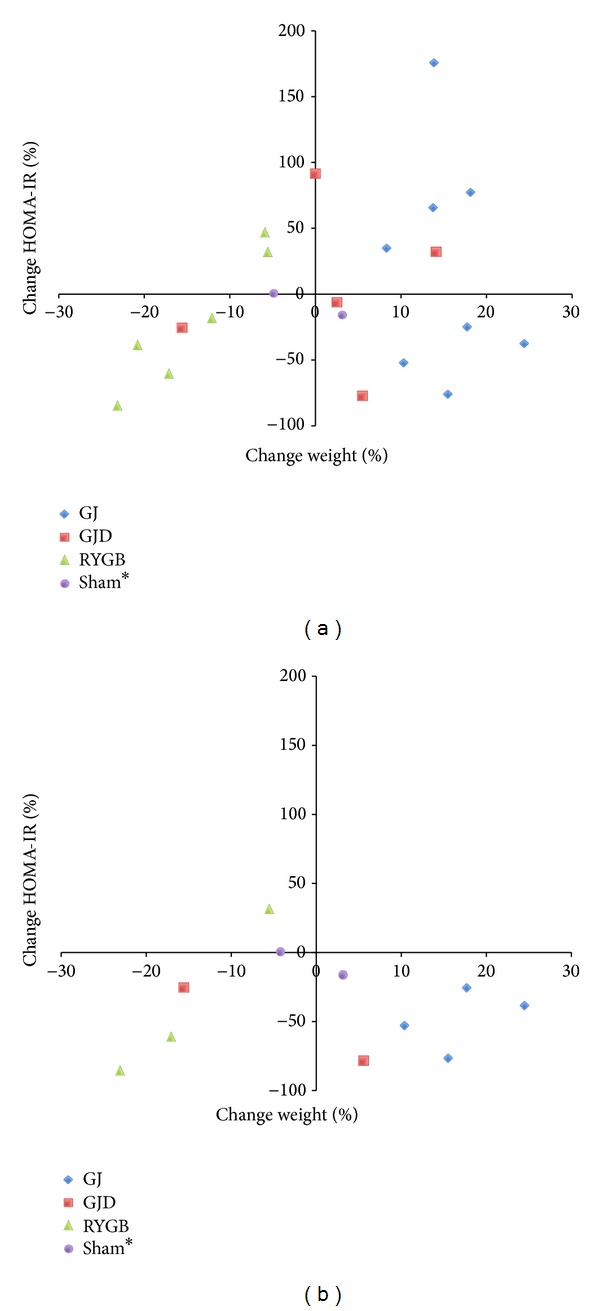
(a) Change in body weight and HOMA-IR by operation. Scatterplot shows percent change in weight and HOMA-IR 8 weeks postoperatively. ∗Sham pig values are at 2 weeks, as 8-week data was unavailable. (b) Change in body weight and HOMA-IR among high HOMA-IR pigs only. Percent change in weight and HOMA-IR 8 weeks postoperatively in pigs with above median baseline HOMA-IRs. ∗Sham pig values are at 2 weeks, as 8-week data was unavailable.

The percentage change in weight and HOMA-IR for each pig 8 weeks after their respective operations are displayed in Figure 2(a). In the GJ group, all pigs gained weight (+15 ± 5%, *P* < 0.001), with no net change in HOMA-IR. Pigs that underwent GJD demonstrated no change of either weight (−0.2 ± 10%, *P* = 0.91) or HOMA-IR (+3 ± 63%, *P* = 0.53) throughout the study and were distributed in all four quadrants of the scatter plot. In contrast with the other procedure groups, all RYGB pigs lost weight during the study period (−14 ± 7%, *P* = 0.002) and the majority (66%) showed a trend towards improvement in insulin resistance.

When evaluating the more insulin-resistant pigs prior to surgery (above median baseline HOMA-IR), almost all pigs demonstrated a postoperative decrease in IR (Figure 2(b)). This improvement across GI procedures was not dependent on weight loss, as half of pigs actually gained weight despite experiencing a drop in HOMA-IR.

### 3.4. Insulin and Glucose AUCs

**Figure 3:**
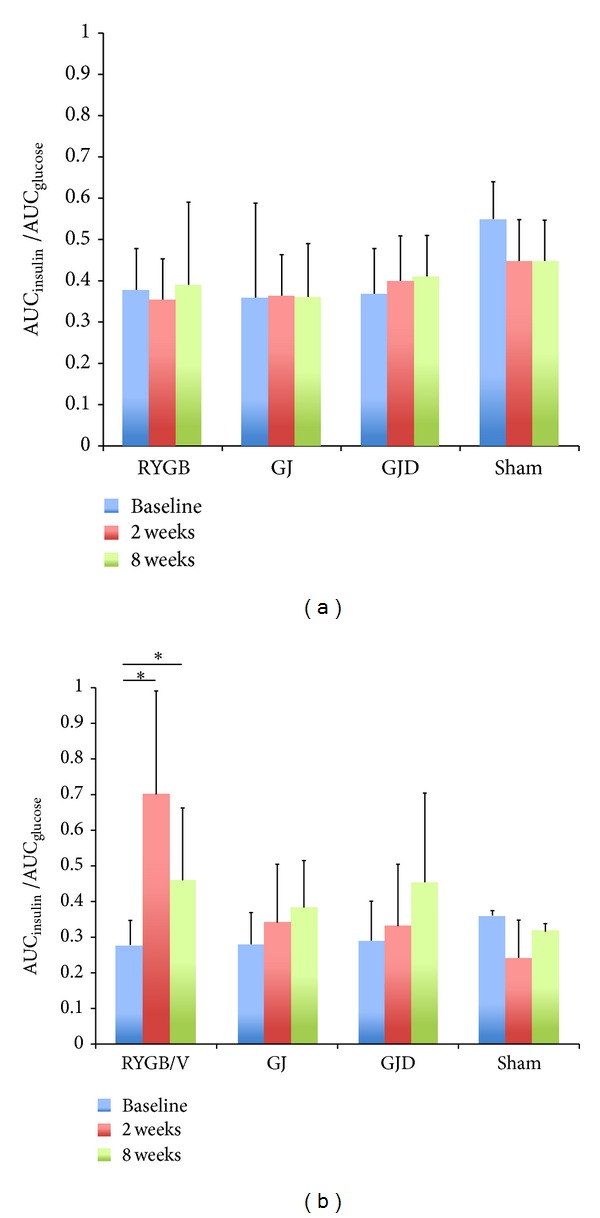
(a) IVGTT AUC_insulin_/AUC_glucose_. Pre- and postoperative IVGTT AUC_insulin_/AUC_glucose_ for RYGB, GJ, GJD, and Sham procedures. (b) MTT AUC_insulin_/AUC_glucose_. Pre- and postoperative MTT AUC_insulin_/AUC_glucose_ for RYGB, GJ, GJD, and Sham procedures. “∗indicates” statistical significance (*P* < 0.05).

For IVGTT, there was no statistically significant difference in AUC_insulin_, AUC_glucose_, or AUC_insulin_/AUC_glucose_ ratio (Figure 3(a)) after 2 or 8 weeks, when compared with baseline values within each procedure, nor between procedures. During MTT (Figure 3(b)), AUC_insulin_/AUC_glucose_ in pigs undergoing RYGB was higher both at 2 weeks (0.7 ± 0.29, *P* = 0.015) and 8 weeks (0.46 ± 0.2, *P* = 0.042) compared with baseline values (0.28 ± 0.07). RYGB was the only procedure associated with a significant increase in AUC_insulin_/AUC_glucose_ during MTT.

## 4. Discussion

The availability of a large animal model to study the mechanisms of antidiabetes effects rendered by RYGB is critical. Identifying the mechanisms of improvement in IR and diabetes observed after bariatric surgery should lay the foundation for the development of targeted, less invasive therapies in the future. Evaluating the molecular pathways activated after RYGB requires evaluation of specific components of the procedure that cannot be easily or ethically performed in humans. Particularly given the limitations of domestic pig and small animal models [36, 37], Ossabaw swine present a more human-like large animal model [15, 17–19, 21], in which long-term survival GI operations can be performed. The robust propensity to obesity in Ossabaw miniature swine clearly makes this breed superior to other laboratory animal swine, such as the Yucatan miniature pig, which show minimal obesity and complete absence of insulin resistance after long-term, high-calorie diets [19, 38]. The anatomy of the porcine upper GI tract, especially the stomach and its neural enervation, is far more similar to corresponding human anatomy than are mouse or rat models. In this report, we highlight not only the unique opportunity that this model affords, but also some important limitations for other investigators to consider.

The majority of Ossabaw swine clearly develop insulin resistance when exposed to a high-fat, high-calorie diet (Figure 1). However, although the overall difference between high-calorie-diet and regular diet pigs was statistically significant, these animals constitute a diverse population, with only ~70% developing insulin resistance, which marginally correlated to increased weight. This is consistent with human epidemiological data indicating that human obesity is itself a heterogeneous condition, with only about one-third of obese persons developing diabetes [39]. Because only a subgroup of obese Ossabaws exhibited deranged glucose homeostasis, future studies of insulin resistance should be performed in animals based on preoperative biochemical testing and validation of insulin resistance, not merely preoperative weight, as was done in this study.

Despite the fact that GI operations were performed in these pigs without consideration of preoperative HOMA-IR, important conclusions can still be gleaned from these data. All GJ pigs gained weight postoperatively (Figure 2(a)), indicating that exclusion of nutrient exposure to the duodenum and proximal jejunum does not, in isolation, promote weight loss in this animal model. Similarly, GJD pigs showed a mixed picture of weight loss/gain after surgery, with no significant change overall in the group. Perhaps for the same reason, when measured across all pigs, none of the operations improved HOMA-IR but pigs with higher baseline insulin resistance experienced a consistent decrease in HOMA-IR after GJ and GJD, regardless of their change in weight (Figure 2(b)). The fact that these pigs demonstrated improved IR despite gaining weight highlights the weight-independent nature of improved IR after GI surgery and is consistent with data from human studies [40]. This also implicates the role of distal nutrient delivery in IR improvement, as both operations deliver nutrients to the jejunum, but only GJD obligatorily bypasses the distal foregut.

The failure of these procedures to positively affect IR in below-median baseline HOMA-IR pigs is an important observation, particularly for investigators who choose to pursue this model in the future. This potentially reflects a “floor effect” of trying to improve HOMA-IR that is already low for that group. Alternatively, this finding could mirror data in some human studies suggesting that diabetes worsens in certain low-BMI or less-diabetic patients after bariatric surgery [41], although the true etiology is unclear and requires further study.

Across all pigs, RYGB was the only procedure to induce both weight loss and a reduction in HOMA-IR in the majority of pigs, regardless of baseline HOMA-IR (Figure 2(a)). Elimination of the gastric reservoir in RYGB is likely the main cause of sustained weight loss, as these pigs also exhibited the largest reduction in food intake of any operation, including GJD, which replicates a RYGB-like proximal intestinal bypass. Although there was a single high-baseline HOMA-IR pig whose insulin resistance did not benefit from the operation, it is important to note that two low-baseline HOMA-IR pigs also experienced an improvement in insulin resistance, an event observed in no other operation. This potentially underscores the ability of RYGB to normalize glucose homeostasis across a more diverse group of obese pigs, consistent with its substantial efficacy in humans.

The IVGTT and MTT data provide insight into the importance of altered nutrient delivery to the physiologic changes that occur after bariatric surgery. We observed a significant rise in AUC_insulin_/AUC_glucose_ in the RYGB cohort during MTT, despite no change during IVGTT in the same pigs. Although changes in AUC_insulin_/AUC_glucose_ have been reported during IVGTT after RYGB [42], it is reasonable to expect greater augmentation of insulin release after nutrient delivery directly to the distal intestine (as in MTT), rather than via a parenteral glucose load, particularly if the “lower intestinal hypothesis” holds true. This could be mediated through augmented secretion of nutrient-stimulated distal intestinal hormones, such as the incretin GLP-1, and/or via a yet undiscovered mechanism. The nonstatistically significant rise in AUC_insulin_/AUC_glucose_ in GJ and GJD pigs is important because these procedures also provide distal nutrient delivery. A key to further defining the interaction between proximal nutrient diversion and distal delivery could be the use of the vertical sleeve gastrectomy procedure, which removes 80% of the gastric mucosa but does not deliver nutrients directly to the jejunum, as in RYGB. This could isolate the effect of the stomach on glucose metabolism to determine if gastric exclusion is sufficient to obtain the observed insulin response, allowing us to further understand the underlying mechanisms of RYGB.

This study has several important limitations that must be considered. The relatively high mortality in the surgical intervention groups reduced the number of animals available for biochemical analysis. While the operative complication rate fell dramatically as the author's experience progressed, omitted animals reduced the statistical power of the remaining cohorts. Additionally, due to a technical error, the two control Sham animals were unable to contribute 8-week blood data, limiting their utility as negative controls. The unexpected finding that only two-thirds of high-calorie-fed animals developed insulin resistance further reduced available data, even in pigs that underwent a GI rearrangement. While this discovery is encouraging as it mimics the heterogeneous human response to obesity, it requires investigators who may use this model in the future to carefully identify insulin-resistant animals prior to randomization and treatment.

## 5. Conclusions

Ossabaw swine exhibit both obesity-dependent and obesity-independent IR and appear to be an effective model assisting in the evaluation of the effect of bariatric surgery on body weight and glucose homeostasis. RYGB, GJ, and GJD result in variable weight loss and improved insulin resistance, especially in pigs with baseline elevated IR. RYGB was associated with significant postoperative elevations in AUC_insulin_/AUC_glucose_ not observed after GJ and GJD, suggesting a combination of upper and lower gut mechanisms. Future studies isolating the effect of nutrient exposure to specific portions of the gastric mucosa and distal intestine should help to further elucidate these effects.

## References

[1] Sjostrom L, Narbro K, Sjostrom CD (2007). Effects of bariatric surgery on mortality in Swedish obese subjects. *The New England Journal of Medicine*.

[2] Buchwald H, Estok R, Fahrbach K (2009). Weight and type 2 diabetes after bariatric surgery: systematic review and meta-analysis. *The American Journal of Medicine*.

[3] Schauer PR, Burguera B, Ikramuddin S (2003). Effect of laparoscopic Roux-en Y gastric bypass on type 2 diabetes mellitus. *Annals of Surgery*.

[4] Fried M, Ribaric G, Buchwald JN, Svacina S, Dolezalova K, Scopinaro N (2010). Metabolic surgery for the treatment of type 2 diabetes in patients with BMI <35 kg/m2: an integrative review of early studies. *Obesity Surgery*.

[5] Jackness C, Karmally W, Febres G (2013). Very low-calorie diet mimics the early beneficial effect of Roux-en-Y gastric bypass on insulin sensitivity and beta-cell function in type 2 diabetic patients. *Diabetes*.

[6] Lindqvist A, Spégel P, Ekelund M (2013). Effects of ingestion routes on hormonal and metabolic profiles in gastric-bypassed humans. *The Journal of Clinical Endocrinology & Metabolism*.

[7] Thaler JP, Cummings DE (2009). Minireview: hormonal and metabolic mechanisms of diabetes remission after gastrointestinal surgery. *Endocrinology*.

[8] Kohli R, Setchell KDR, Kirby M (2013). A surgical model in male obese rats uncovers protective effects of bile acids post-bariatric surgery. *Endocrinology*.

[9] Ryan KK, Tremaroli V, Clemmensen C (2014). FXR is a molecular target for the effects of vertical sleeve gastrectomy. *Nature*.

[10] Sweeney TE, Morton JM (2013). The human gut microbiome: a review of the effect of obesity and surgically induced weight loss. *JAMA Surgery*.

[11] Liou AP, Paziuk M, Luevano J, Machineni S, Turnbaugh PJ, Kaplan LM (2013). Conserved shifts in the gut microbiota due to gastric bypass reduce host weight and adiposity. *Science Translational Medicine*.

[12] Breen DM, Rasmussen BA, Kokorovic A, Wang R, Cheung GWC, Lam TKT (2012). Jejunal nutrient sensing is required for duodenal-jejunal bypass surgery to rapidly lower glucose concentrations in uncontrolled diabetes. *Nature Medicine*.

[13] Saeidi N, Meoli L, Nestoridi E (2013). Reprogramming of intestinal glucose metabolism and glycemic control in rats after gastric bypass. *Science*.

[14] Varga O, Harangi M, Olsson IAS, Hansen AK (2010). Contribution of animal models to the understanding of the metabolic syndrome: a systematic overview. *Obesity Reviews*.

[15] Sturek MAM, Wenzel J, Byrd JP, Swindle MM (2007). Ossabaw island miniature swine: cardiometabolic syndrome assessment. *Swine in the Laboratory: Surgery, Anesthesia, Imaging, and Experimental Techniques*.

[16] Flum DR, Devlin A, Wright AS (2007). Development of a porcine Roux-en-Y gastric bypass survival model for the study of post-surgical physiology. *Obesity Surgery*.

[17] Wangsness PJ, Martin RJ, Gahagan JH (1977). Insulin and growth hormone in lean and obese pigs. *The American Journal of Physiology—Heart and Circulatory Physiology*.

[18] Edwards JM, Neeb ZP, Alloosh MA (2010). Exercise training decreases store-operated Ca^2+^entry associated with metabolic syndrome and coronary atherosclerosis. *Cardiovascular Research*.

[19] Neeb ZP, Edwards JM, Alloosh M, Long X, Mokelke EA, Sturek M (2010). Metabolic syndrome and coronary artery disease in ossabaw compared with yucatan swine. *Comparative Medicine*.

[20] Lee L, Alloosh M, Saxena R (2009). Nutritional model of steatohepatitis and metabolic syndrome in the Ossabaw miniature swine. *Hepatology*.

[21] Etherton TD, Kris-Etherton PM (1980). Characterization of plasma lipoproteins in swine with different propensities for obesity. *Lipids*.

[22] Thaler JP, Cummings DE (2008). Metabolism: food alert. *Nature*.

[23] Rubino F, Forgione A, Cummings DE (2006). The mechanism of diabetes control after gastrointestinal bypass surgery reveals a role of the proximal small intestine in the pathophysiology of type 2 diabetes. *Annals of Surgery*.

[24] Cummings DE, Weigle DS, Scott Frayo R (2002). Plasma ghrelin levels after diet-induced weight loss or gastric bypass surgery. *The New England Journal of Medicine*.

[25] Cummings DE, Shannon MH (2003). Ghrelin and gastric bypass: is there a hormonal contribution to surgical weight loss?. *Journal of Clinical Endocrinology and Metabolism*.

[26] Cummings DE, Overduin J, Shannon MH, Foster-Schubert KE (2005). Hormonal mechanisms of weight loss and diabetes resolution after bariatric surgery. *Surgery for Obesity and Related Diseases*.

[27] Cummings DE, Overduin J, Foster-Schubert KE, Carlson MJ (2007). Role of the bypassed proximal intestine in the anti-diabetic effects of bariatric surgery. *Surgery for Obesity and Related Diseases*.

[28] Cummings DE, Overduin J, Foster-Schubert KE (2004). Gastric bypass for obesity: mechanisms of weight loss and diabetes resolution. *Journal of Clinical Endocrinology and Metabolism*.

[29] Cummings DE, Flum DR (2008). Gastrointestinal surgery as a treatment for diabetes. *Journal of the American Medical Association*.

[30] Cummings DE (2005). Gastric bypass and nesidioblastosis—too much of a good thing for islets?. *The New England Journal of Medicine*.

[31] Courcoulas AP, Flum DR (2005). Filling the gaps in bariatric surgical research. *Journal of the American Medical Association*.

[32] Association APoEAVM (2001). 2000 Report of the AVMA panel on euthanasia. *Journal of the American Veterinary Medical Association*.

[33] National Research Council (2011). *(US) Committee for the Update of the Guide for the Care and Use of Laboratory Animals*.

[34] Lombardo C, Damiano G, Cassata G (2010). Surgical vascular access in the porcine model for long-term repeated blood sampling. *Acta Biomedica*.

[35] Matthews DR, Hosker JP, Rudenski AS, Naylor BA, Treacher DF, Turner RC (1985). Homeostasis model assessment: insulin resistance and *β*-cell function from fasting plasma glucose and insulin concentrations in man. *Diabetologia*.

[36] Kirchner H, Guijarro A, Meguid MM (2007). Is a model useful in exploring the catabolic mechanisms of weight loss after gastric bypass in humans?. *Current Opinion in Clinical Nutrition and Metabolic Care*.

[37] Arner P (2005). Resistin: yet another adipokine tells us that men are not mice. *Diabetologia*.

[38] Otis CR, Wamhoff BR, Sturek M (2003). Hyperglycemia-induced insulin resistance in diabetic dyslipidemic Yucatan swine. *Comparative Medicine*.

[39] Adult Obesity Facts http://www.cdc.gov/obesity/data/adult.html.

[40] Cohen RV, Pinheiro JC, Schiavon CA, Salles JE, Wajchenberg BL, Cummings DE (2012). Effects of gastric bypass surgery in patients with type 2 diabetes and only mild obesity. *Diabetes Care*.

[41] DiGiorgi M, Rosen DJ, Choi JJ (2010). Re-emergence of diabetes after gastric bypass in patients with mid- to long-term follow-up. *Surgery for Obesity and Related Diseases*.

[42] Lindqvist A, Spegel P, Ekelund M (2014). Gastric bypass improves ss-cell function and increases beta-cell mass in a porcine model. *Diabetes*.

